# Effects of mode of administration (MOA) on the measurement properties of the EORTC QLQ-C30: a randomized study

**DOI:** 10.1186/1477-7525-8-35

**Published:** 2010-03-30

**Authors:** Chad M Gundy, Neil K Aaronson

**Affiliations:** 1 Division of Psychosocial Research and Epidemiology, The Netherlands Cancer Institute, 121 Plesmanlaan, 1066 CX Amsterdam, The Netherlands

## Abstract

**Background:**

While modern electronic data collection methods (e.g., computer touch-screen or web-based) hold much promise, most current studies continue to make use of more traditional data collection techniques, including paper-and-pencil administration and telephone interviews. The present randomized trial investigated the measurement properties of the EORTC QLQ-C30 under three different modes of administration (MOA's).

**Methods:**

A heterogeneous sample of 314 cancer patients undergoing treatment at a specialized treatment center in Amsterdam were randomized to one of three MOA's for the QLQ-C30: paper-and-pencil at home via the mail, telephone interview, and paper-and-pencil at the hospital clinic. Group differences in internal consistency reliabilities (Cronbach's alpha coefficient) for the scale scores were compared. Differences in mean scale scores were also compared by means of ANOVA, with adjustment for potential confounders.

**Results:**

Only one statistically significant, yet minor, difference in Cronbach's alpha between the MOA groups was observed for the Role Functioning scale (all 3 alphas >0.80). Significant differences in group means -after adjustment- were found for the Emotional Functioning (EF) scale. Patients completing the written questionnaire at home had significantly lower levels of EF as compared to those interviewed via the telephone; EF scores of those completing the questionnaire at the clinic fell in-between those of the other two groups. These differences, however, were small in magnitude.

**Conclusions:**

MOA had little effect on the reliability or the mean scores of the EORTC QLQ-C30, with the possible exception of the EF scale.

## Background

Health-related quality of life (HRQoL) questionnaires can be administered using a variety of methods, including face-to-face or telephone interviews, pencil and paper, computer touch-screen, or web-based. However, not all researchers may have equal access to all modes of administration (MOA). For example, despite the attractiveness of high-tech electronic methods, none of the 107 abstracts cited in PubMed for 2007 concerning the EORTC QLC-C30 HRQoL questionnaire reported having used a computer for data collection.

In addition, various MOA may not be equally practical for all respondents. For example, lack of language or computer skills may preclude the use of written questionnaires, whether pencil and paper or computer-based. It may also be sometimes necessary to combine multiple modes of administration in the same study, for example when conducting longitudinal research or combining data from various sources.

For these reasons, it is important to consider whether the measurement characteristics of various MOA's are equivalent, because, if this is not the case, then it would be difficult to compare outcomes across MOA's within or between studies. Many studies of varying designs, sizes, populations, and instruments have considered this issue, with generally similar results [1-9]. Namely, the effects of MOA on questionnaire measurement characteristics are generally not large. However, only two studies have investigated the effect of MOA on the EORTC QLQ-C30, one of the most widely used HRQoL questionnaires in oncology [10-15]. In a large (N = 855) observational study, Cheung et al. [14] investigated the effect of two MOA's, in-clinic interview with in-clinic paper-and-pencil, on the measurement properties on 4 multi-item scales of the QLQ-C30. Velikova et al. [15] used all 15 scales of the QLQ-C30 in a randomized, cross-over study of 149 patients, comparing in-clinic touch-screen with in-clinic paper & pencil administration. Despite their differences and limitations, these two studies each found several small, yet statistically significant, differences in scale mean scores as a function of MOA's (approximately 3-7 points on a 100 point scale). Both studies flagged the Emotional Functioning Scale as being potentially problematical.

The purpose of the current study was to investigate, in a controlled, randomized setting, the measurement characteristics of the EORTC QLQ C-30 under a variety of different, conventional MOA's.

## Methods

### Study Sample

The study sample employed in the current analysis was composed of participants in a study conducted by te Velde and colleagues that evaluated various instruments for HRQoL assessment in oncology [15,16].

### Patients

The patient sample was composed of individuals with a variety of cancer diagnoses (primarily breast, colorectal, and lung) with various disease stages (local, loco-regional, or metastasized) who attended the Netherlands Cancer Institute/Antoni van Leeuwenhoek Hospital for treatment. The data used in the current analysis were collected approximately 4 months after start of radio- or chemotherapy, during the third measurement wave (T3) in a longitudinal study.

Exclusion criteria included a life expectancy of less than 4 months, too ill to participate, participation in a concurrent HRQoL study, less than 18 years of age, and a lack of basic proficiency in Dutch. No restrictions were made with regard to age or performance status. Eligible patients received a full, verbal and written explanation of the purpose and procedures of the study. The study was approved by the local ethics committee, and written informed consent was obtained from all participating patients.

#### Patient Characteristics

A number of variables, which were possibly relevant for the quality of patient ratings of HRQoL, were measured for the purpose of describing the sample of patients, as well as for assessing the quality of the randomization into three groups. Characteristics of the patients included: indicators of health (i.e., the Karnofsky Performance Status scale [17]), treatment and disease characteristics, comorbidity, sociodemographic data, and the EORTC QLQ-C30 questionnaire data collected during the previous (in-clinic) measurement wave at T2.

### Procedure

To assess the impact of different MOA's on the measurement performance of the EORTC QLQ-C30, patients were randomly assigned (with equal probabilities), during the first measurement wave of the study at T1, to one of three groups during the third measurement wave at T3: in-clinic written self-administration; telephone-based interviewer-administration, or mailed written self-administration.

### Health-related quality of life (HRQoL) assessment

HRQoL was assessed with the European Organization for Research and Treatment of Cancer (EORTC) Quality of Life Questionnaire (QLQ-C30 (version 2.0)) [10-13]. It includes 5 functional scales (physical, role, cognitive, emotional, and social), 3 symptom scales (fatigue, nausea and vomiting, and pain), 6 single items (dyspnea, insomnia, anorexia, constipation, diarrhea, and financial impact), and 1 global quality of life scale. The questionnaire employs a one-week time frame and a mix of dichotomous response categories ("yes/no"), 4-point Likert-type response scales (ranging from "not at all" to "very much"), and 7-point response scales (numbered visual analogue scales). The scoring procedures recommended by the EORTC [13] were used. All scale and single item scores of the QLQ-C30 were linearly transformed to a 0 to 100 scale. For the functioning scales, higher scores represent a better level of functioning; for the symptom measures, a higher score corresponds to a higher level of symptomology.

The QLQ-C30 has been shown to be reliable and valid in a range of patient populations and treatment settings. Across a number of studies, internal consistency estimates (Cronbach's coefficient α) of the multi-item scales exceeded or approached 0.70 [12]. Test-retest reliability coefficients have been found to range between 0.80 and 0.90 for most multi-item scales and single items [18]. Tests of validity have shown the QLQ-C30 to be responsive to meaningful between-group differences (e.g., local vs. metastatic disease, active treatment vs. follow-up) and changes in clinical status over time [10,12].

### Statistical Methods

Mean scores and standard deviations for the QLQ-C30 scales, as well as for the characteristics of the patients were calculated. The internal consistency of the multi-item scales of the QLQ-C30 was assessed by Cronbach's coefficient alpha [19,20].

Differences in scale/item means were tested by means of analysis of co-variance (ANCOVA), which allowed adjustment for possible confounders. To examine the magnitude of any observed difference between MOA's, mean difference scores between groups were then standardized by dividing them by the pooled standard deviation, in order to estimate an effect size [21]. Following Cohen [21], effect sizes of 0.20, 0.50, and 0.80 were considered small, medium, and large, respectively. Osoba et al. [22] determined that a difference of 10 or fewer points on the (re-scaled) QLQ-C30 scales could be viewed as being "small".

Levene's test for the equality of variances between groups was also calculated. Finally, multiple analysis of (co-)variance provided a multivariate test of differences between groups, with adjustment for possible confounders. For all tests, the type I error (alpha) significance level was set at 0.05.

## Results

### Sample accrual (Figure 1)

**Figure 1:**
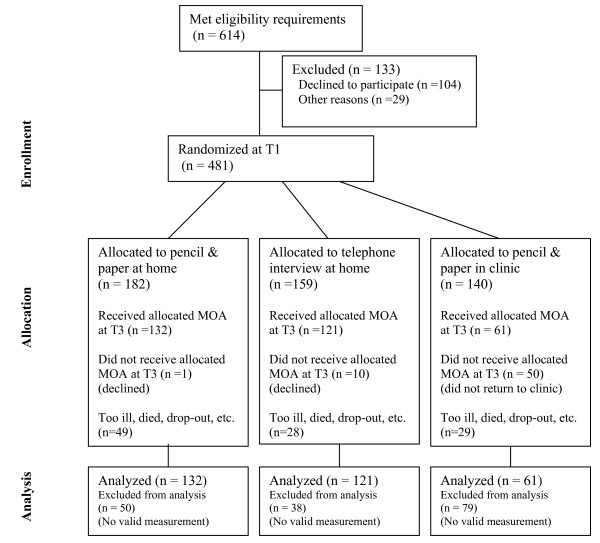
**Results of Patient accrual and randomization**.

During the study period, 614 patients who met the eligibility criteria were invited to participate in the study, of whom 483 (79%) accepted at T1. Reasons for declining study participation included: (a) the study was perceived as too emotionally burdensome (n = 54); (b) perceived lack of time (n = 22); (c) lack of interest (n = 18); or (d) being too ill (n = 10). The remaining 29 patients had a variety of other reasons. Patients declining participation were, on average, older (mean age 65 years vs. 57 years), were less frequently married (59% vs. 76%), and more often had compulsory education only (91% vs. 82%), than those who participated.

Of the 483 patients initially enrolled in the study, and randomized at the first assessment point T1, 375 (78%) remained available for the actual measurement at T3, which was used for the present analysis. The primary reasons for patient attrition were severe illness (*n *= 36) or death (*n *= 35). Patients lost to follow-up were more likely to have metastatic disease, and their KPS was 10 to 30 points lower than patients who continued participation. The average time between Tl and T3 was 128 days.

However, after randomization, 11 patients declined to participate in the MOA condition to which they were assigned, and 50 patients randomized to the in-clinic condition did not attend the hospital for a follow-up visit that coincided with this -third- assessment point. These patients were also excluded from further analysis.

### Statistical Power

We determined that the present sample size would be able to detect a "medium" effect size for differences in means (d = 0.50) between two groups with a power exceeding 90%, (assuming a two-sided test with a significance of 5%) [21].

### Sample characteristics (Tables 1 and 2)

**Table 1:** Patient sample characteristics (n = 314) for 3 MOA groups

Individual Characteristics	Paper & pencil at home (n = 132)	Telephone (n = 121)	Paper & pencil in-clinic (n = 61)	
	**Means (s.d.)**	**Means (s.d.)**	**Means (s.d.)**	**Sig.**
				
Age	56.6(12.9)	57.0(12.0)	54.1(11.5)	.30
KPS	77.4(14.4)	78.1(13.7)	78.0(15.0)	.93
				
	**N(%)**	**N(%)**	**N(%)**	
				
Sex (%)				
Male	48(36%)	47(39%)	16(27%)	.23
				
Marital Status				
Single	11(8%)	9(8%)	6(10%)	.92
Married	99(75%)	97(81%)	44(73%)	
Divorced	10(8%)	7(6%)	5(8%)	
Widowed	12(9%)	7(6%)	5(8%)	
				
Education				
<10 years	70(53%)	69(58%)	32(53%)	.21
10-15 years	39(30%)	35(29%)	12(20%)	
>15 years	23(17%)	16(13%)	16(27%)	
				
Employed				
Yes	50(38%)	41(35%)	19(32%)	.70
				
Stage of Disease*				
Local/regional	86(66%)	73(61%)	28(47%)	.04
				
Treatment**				
Chemotherapy	56(42%)	50(41%)	44(73%)	<.00
Radiotherapy	70(53%)	67(55%)	15(25%)	
RT+CT/other	6(5%)	4(3%)	1(2%)	
				
Comorbidity				
Yes	74%	74%	69%	.76
				
Primary Site				
Breast	48(36%)	49(41%)	32(53%)	.11
Colorectal	38(29%)	31(26%)	10(17%)	
Lung	36(27%)	29(24%)	9(15%)	
Other	10(8%)	12(10%)	9(15%)	

* p < 0.05 ** p < 0.01

**Table 2:** Patient sample characteristics (n = 314) for 3 MOA groups at Pretest *(T2)*

**QLQ-C30** **at pre-test (T2)**	**Paper & pencil at home** **(n = 132)**		**Telephone** **(n = 121)**		**Paper & pencil in-clinic** **(n = 61)**		
	
	**Mean (s.d.)**	**%** **floor/ceiling**	**Mean (s.d.)**	**%** **floor/ceiling**	**Mean (s.d.)**	**%** **floor/ceiling**	**Sig.**
	
Physical function	62.0(28.9)	2%/23%	69.9(23.0)	2%/26%	66.8(24.2)	2%/23%	.052
Role function	59.2(31.3)	4%/27%	67.9(28.3)	5%/36%	66.9(28.6)	12%/35%	.050
Cognitive function*	80.0(18.4)	0%/43%	82.5(18.9)	1%/50%	87.2(14.4)	0%/39%	.038
Emotional function	74.4(21.0)	1%/20%	77.7(19.6)	1%/36%	78.6(18.7)	0%/26%	.294
Social function	76.5(27.6)	2%/52%	82.8(20.5)	1%/60%	81.7(22.7)	2%/51%	.103
GlobalHealth/QoL	61.5(22.0)	1%/6%	67.2(19.5)	1%/12%	66.1(19.6)	0%/8%	.078
Fatigue	45.6(26.0)	13%/3%	38.2(23.5)	21%/3%	40.1(26.9)	15%/3%	.060
Nausea/vomitig	14.1(21.3)	69%/1%	10.4(19.0)	76%/1%	13.4(18.5)	62%/2%	.321
Pain**	30.5(29.9)	30%/3%	24.7(26.2)	45%/4%	14.8(20.4)	46%/0%	.001
Dyspnea	20.1(27.9)	55%/4%	18.1(24.0)	55%/0%	18.0(24.0)	51%/2%	.785
Insomnia	30.0(33.8)	52%/8%	29.4(31.8)	63%/2%	21.9(28.5)	57%/3%	.226
Anorexia	24.2(29.8)	72%/2%	19.1(29.3)	72%/5%	22.2(29.9)	66%/2%	.392
Constipation	11.8(23.8)	83%/2%	7.8(19.2)	87%/1%	12.0(21.1)	82%/0%	.268
Diarrhea	10.8(21.7)	85%/1%	11.7(22.7)	85%/0%	4.4(13.0)	87%/0%	.074
Financial	8.2(19.0)	82%/1%	5.6(13.9)	89%/2%	3.3(11.7)	90%/0%	.117

* p < 0.05 ** p < 0.01

Characteristics of the patients in each of the three MOA groups are presented in Table 1. Pre-test HRQoL measurements, taken at T2, are presented in Table 2. Of those patients remaining in the study at T3, very few data were missing, not exceeding 3% for any of the QLQ-C30 scales for any of the three conditions (data not shown). Mainly due to the loss of the 50 patients randomized to the in-clinic condition, there was an imbalance in the number of patients per group, and in the distribution of stage of disease, type of treatment, and several previous QLQ-C30 scale scores between the three groups. These 50 dropout-patients differed from the patients remaining in the in-clinic condition primarily in terms of type of treatment (p < 0.05, after adjustment for other predictors).

### Internal Consistency of the QoL proxy scales (Table 3)

**Table 3:** Cronbach's alpha's for multi-item Scales for three MOA groups

EORTC QLQ-C30 Multi-item Scales	Paper & pencil at home (N = 132)	Telephone (N = 121)	Paper & pencil in-clinic (N = 61)
Physical function	0.69	0.71	0.69
Role function* ^&^	0.87	0.80	0.93
Cognitive function	0.57	0.64	0.57
Emotional function	0.86	0.86	0.84
Social function	0.78	0.64	0.81
Global health/QoL	0.84	0.84	0.89
Fatigue	0.87	0.87	0.88
Nausea/vomiting	0.73	0.75	0.64
Pain	0.86	0.87	0.79

* p < 0.05

** p < 0.01

& The significant difference in this comparison is between the telephone versus the in-clinic Paper & Pencil condition

Cronbach's alpha's for the multi-item scales for each group were generally adequate (i.e., > 0.70) in the large majority of cases. The consistent exception was the Cognitive Functioning scale; something that has been observed in many other studies. There was a significant difference between the in-clinic paper-and-pencil and the telephone conditions for the Role Functioning (RF) scale, even though this scale performed rather well (alpha > = 0.8) for all three conditions.

### Mean QLQ-C30 scale score differences (Table 4)

**Table 4:** Adjusted# Means (+s.e.) for three MOA groups

EORTC QLQ-C30 All Scales	Paper & pencil at home (N = 132)	Telephone (N = 121)	Paper & Pencil in clinic (N = 61)	
	**Mean (s.e.)**	**Mean (s.e.)**	**Mean (s.e.)**	**Sig.#**
				
Physical function	68.9(2.9)	67.1(3.0)	65.3(3.6)	.47
Role function	61.4(3.3)	62.2(3.3)	60.4(3.9)	.86
Cognitive function	86.2(2.7)	86.0(2.8)	84.8(3.3)	.87
Emotional function* ^&^	74.1(2.7)	79.5(2.8)	75.0(3.3)	.04
Social function	79.1(3.1)	79.2(3.2)	76.9(3.8)	.76
Global Health/QoL	64.7(2.6)	66.0(2.7)	64.5(3.2)	.79
Fatigue	41.0(3.2)	41.9(3.2)	45.3(3.8)	.42
Nausea/vomiting	6.9(2.9)	6.2(3.0)	7.5(3.5)	.90
Pain	33.6(3.6)	33.1(3.7)	26.0(4.4)	.11
Dyspnea	24.4(3.4)	26.4(3.5)	28.6(4.2)	.48
Insomnia	30.5(4.3)	22.3(4.4)	27.3(5.2)	.06
Anorexia	8.7(3.9)	13.4(4.0)	13.0(4.7)	.29
Constipation	4.2(3.1)	4.8(3.1)	1.7(3.7)	.61
Diarrhea	6.2(2.5)	6.4(2.6)	5.7(3.1)	.97
Financial	10.4(2.9)	8.7(2.9)	5.8(3.5)	.30

*p < 0.05 ** p < 0.01

# adjusted for covariates in Tables 1 and 2

& overall effect size for Emotional Function scale are 0.16 for overall effect (Cohen's f, based on partial eta squared) and 0.31, 0.14, 0.19 (Cohen's d) for the "telephone vs. paper & pencil at home", the "paper & pencil at home vs. pencil & paper in-clinic", and the "telephone vs. paper & pencil in-clinic" pairs of conditions, respectively.

The adjusted means and standard errors of the three MOA groups for each of the 15 QLQ-C30 scales are presented in Table 4. After adjustment for the possible confounders shown in Table 1 and 2, significant group differences were found only for Emotional Functioning (EF). The telephone condition had the highest EF, and the paper-and-pencil at-home condition the lowest. The un-adjusted mean difference between these two conditions was approximately 6 points, the adjusted mean difference being only 5.4 points. The pair-wise Cohen's d's for the "telephone vs. paper & pencil at home", the "paper & pencil at home vs. pencil & paper in-clinic", and the "telephone vs. paper & pencil in-clinic" conditions were 0.31, 0.14, 0.19, respectively. These results qualify the MOA effect for the EF scale as being "small".

An additional analysis was conducted, adding a fourth group of patients to the above analyses of differences between means. This fourth group consisted of the patients who were not available for the in-clinic paper & pencil condition because they did not return to the clinic at T3. These patients were invited to complete the questionnaire in the same manner as the "paper & pencil at home" condition. Results indicated that patients in this fourth group had significantly poorer scores for the EF and SL scales as compared to the" telephone" condition, and did not differ from the original paper & pencil conditions (data not shown).

### Miscellaneous statistical tests

A Levene test for difference in variances between the groups was significant for Pain, Appetite loss, and Financial Difficulties (p < 0.05). A multivariate analysis of variance (Pillai's trace/Wilk's lambda, with adjustment for confounders) found no significant difference (p = 0.40) between the three groups. (Data not presented.)

## Discussion

In this study we investigated several measurement properties of the EORTC QLQ-C30 questionnaire under various MOA's. Despite the widespread use of the EORTC QLQ-C30, only two studies had previously investigated this matter. One large observational study considered 4 of the QLQ-C30 multi-item scales [14], while the other study used a randomized, cross-over design, but with a much smaller sample size, and with only two (in-clinic) conditions [15].

The present study of a heterogeneous population of 375 cancer patients considered three conditions (at-home as well as in-clinic) in a randomized, between-subjects trial.

Remarkably, all three of these studies flag the Emotional Functioning (EF) scale as yielding a small, yet statistically significant difference as a function of MOA, with patients in paper-and-pencil MOA's reporting lower levels of emotional functioning. The present study also found a small, yet significant difference in Cronbach's alpha for the Role Functioning scale; however, the RF scale performed quite adequately for all three conditions.

We suspect that the slightly lower EF scale scores in paper-and-pencil conditions may be related to the "demand characteristics" associated with different MOA's. Specifically, patients, who are encouraged to react quickly and/or who are required to interact with an interviewer, may be stimulated to present more socially desirable responses than those patients allowed to reflect on their level of emotional functioning and whose responses to the questions are not the subject of direct observation. For example, patients are asked in the QLQ-C30 whether they are depressed, which is not a directly observable state, and whose admission might be felt as being potentially stigmatizing.

Many studies of varying designs, sizes, populations, and instruments have considered the issue of measurement characteristics of various MOA, with generally similar results. Namely, while various MOA may differ in costs, completion rates, etc., the effects of MOA on questionnaire measurement characteristics are generally of "small to medium" size, if found at all. This would suggest that one should exercise caution when mixing MOA's while investigating effects of similar magnitudes.

A limitation associated with the present investigation concerns the post-randomization dropout of patients prior to assessment. This occurred primarily in the in-clinic condition. Almost 50% of the patients allocated to this condition did not return to the clinic in time for the present study. This differential drop-out (apparently) lead to group differences in patient characteristics, such as treatment, stage of disease, and pre-randomization HRQoL measures. However, we believe that adjustment for these patient characteristics in the statistical analyses was largely able to correct for these group differences. An additional, sensitivity analysis included these in-clinic dropouts, who were approached via "pencil & paper at home". This analysis re-flagged the EF scale, as well as the SL scale, indicating that the "telephone" MOA yielded a more positive result than pencil & paper conditions (which did not differ from each other). These findings are commensurate with the finding reported above.

A second limitation concerns the use of version 2.0 of the EORTC QLQ-C30. There are, namely, slight differences with the current version 3.0, involving the number of response categories for the Physical Function scales. This might slightly limit the generalizability of these results to users of version 3.0.

## Conclusions

In conclusion, the findings of this investigation indicate that the 3 modes of administration studied here have little effect on the internal consistency or the mean responses on the EORTC QLQ-C30 scales. The exception to this generalization is the Emotional Functioning scale, which exhibited small, yet significant, differences between various administration modes. These results suggest that, with the possible exception of assessment of emotional functioning, there is little reason for concern about the comparison of QLQ-C30 results within or across studies as a function of mode of administration.

## Competing interests

The authors declare that they have no competing interests.

## Authors' contributions

NA conceived of the study, and participated in its design and coordination and helped to draft the manuscript. CG participated in the design of the study, performed the statistical analysis, and drafted the manuscript. All authors read and approved the final manuscript.

## References

[B1] Barry MJ Fowler FJ Chang Y Liss CL Wilson H Stek M Jr The American Urological Association symptom index: does mode of administration affect its psychometric properties? J Urol 1995 154 1056 1059 10.1016/S0022-5347(01)66975-1 7543597

[B2] Weinberger M Oddone EZ Samsa GP Landsman PB Are health-related quality-of-life measures affected by the mode of administration? J Clin Epidemiol 1996 49 135 140 10.1016/0895-4356(95)00556-0 8606314

[B3] Vereecken CA Maes L Comparison of a computer-administered and paper-and-pencil-administered questionnaire on health and lifestyle behaviors J Adolesc Health 2006 38 426 432 10.1016/j.jadohealth.2004.10.010 16549304

[B4] Fouladi RT McCarthy CJ Moller NP Paper-and-pencil or online? Evaluating mode effects on measures of emotional functioning and attachment Assessment 2002 9 204 215 10.1177/10791102009002011 12066835

[B5] Rhodes T Girman CJ Jacobsen SJ Guess HA Hanson KA Oesterling JE Lieber MM Does the mode of questionnaire administration affect the reporting of urinary symptoms? Urology 1995 46 341 345 10.1016/S0090-4295(99)80217-9 7660509

[B6] Wu AW Jacobson DL Berzon RA Revicki DA Horst C van der Fichtenbaum CJ Saag MS Lynn L Hardy D Feinberg J The effect of mode of administration on Medical Outcomes Study health ratings and EuroQol scores in AIDS Quality of Life Research 1997 6 0 10.1023/A:1026471020698 9062436

[B7] Weinberger M Nagle B Hanlon JT Samsa GP Schmader K Landsman PB Uttech KM Cowper PA Cohen HJ Feussner JR Assessing health-related quality of life in elderly outpatients: telephone versus face-to-face administration J Am Geriatr Soc 1994 41 1295 1299 10.1111/j.1532-5415.1994.tb06515.x 7983296

[B8] Jorngarden A Wettergen L von Essen L Measuring health-related quality of life in adolescents and young adults: Swedish normative data for the SF-36 and the HADS, and the influence of age, gender, and method of administration Health Qual Life Outcomes 2006 4 91 10.1186/1477-7525-4-91 17140436 PMC1697805

[B9] Perkins JJ Sanson-Fisher RW An examination of self- and telephone-administered modes of administration for the Australian SF-36 J Clin Epidemiol 1998 51 969 973 10.1016/S0895-4356(98)00088-2 9817114

[B10] Aaronson NK Ahmedzai S Bergman B Bullinger M Cull A Duez NJ Filiberti A Flechtner H Fleishman SB De Haes JC The European Organization for Research and Treatment of Cancer QLQ-C30: a quality-of-life instrument for use in international clinical trials in oncology J Natl Cancer Inst 1993 85 365 376 10.1093/jnci/85.5.365 8433390

[B11] Osoba D Aaronson NK Zee B Modification of the EORTC QLQ-C30 (version 2.0) based on content validity and reliability testing in large samples of patients with cancer Qual Life Res 1997 6 103 108 10.1023/A:1026429831234 9161109

[B12] Aaronson NK Cull A Kaasa S Sprangers MAG Spilker B The European Organization for Research and Treatment of Cancer (EORTC) modular approach to quality of life assessment in oncology: an update Quality of life and pharmacoeconomics in clinical trials 1996 2 Philadelphia: Lippincott-Raven Publishers 179 189

[B13] Fayers PM Aaronson N Bjordal K Groenvold M Curran D Bottomley A on behalf of the EORTC Quality of Life Group EORTC QLQ-C30 Scoring Manual 2001 Brussels: European Organization for Research and Treatment of Cancer

[B14] Cheung YB Goh C Thumboo J Khoo KS Wee J Quality of life scores differed according to mode of administration in a review of three major oncology questionnaires J Clin Epidemiol 2006 59 185 191 10.1016/j.jclinepi.2005.06.011 16426954

[B15] Velikova G Wright EP Smith AB Cull A Gould A Forman D Perren T Sted M Brown J Selby PJ Automated collection of quality-of-life data: a comparisons of paper and computer touch-screen questionnaires J Clin Oncol 1999 17 998 1007 10071295 10.1200/JCO.1999.17.3.998

[B16] te Velde A Sprangers M Aaronson NK Feasibility, psychometric performance, and stability across modes of administration of the CARES-SF Annals of Oncology 1996 7 381 390 8805930 10.1093/oxfordjournals.annonc.a010605

[B17] Karnofsky D Burchenal J MacLeod C The clinical evaluation of chemotherapeutic agents in cancer Evaluation of Chemotherapeutic Agents 1949 New York: Columbia University Press

[B18] Hjermstad MJ Fossa SD Bjordal K Kaasa S Test/retest study of the European Organization for Research and Treatment of Cancer Core Quality-of-Life Questionnaire J CLIN ONCOL 1995 13 1249 1254 7738629 10.1200/JCO.1995.13.5.1249

[B19] Cronbach LJ Coefficient alpha and the internal structure of tests Psychometrika 1951 16 297 334 10.1007/BF02310555

[B20] Lautenschlager GJ ALPHATST: Testing for differences in coefficient alpha Appl Psychol Meas 1989 13 284 10.1177/014662168901300308

[B21] Cohen J Statistical power analysis for the behavioral sciences 1988 Hillsdale, New Yersey: Lawrence Erlbaum Associates

[B22] Osoba D Rodrigues G Myles J Zee B Pater J Interpreting the Significance of Changes in Health-Related Quality-of-Life Scores Journal of Clinical Oncology 1998 16 1 139 144 9440735 10.1200/JCO.1998.16.1.139

